# RNAi–Based Functional Profiling of Loci from Blood Lipid Genome-Wide Association Studies Identifies Genes with Cholesterol-Regulatory Function

**DOI:** 10.1371/journal.pgen.1003338

**Published:** 2013-02-28

**Authors:** Peter Blattmann, Christian Schuberth, Rainer Pepperkok, Heiko Runz

**Affiliations:** 1 Cell Biology and Biophysics Unit, European Molecular Biology Laboratory, Heidelberg, Germany; 2 Molecular Medicine Partnership Unit (MMPU), European Molecular Biology Laboratory, Heidelberg, Germany; 3 Institute of Human Genetics, University of Heidelberg, Heidelberg, Germany; University of Oxford, United Kingdom

## Abstract

Genome-wide association studies (GWAS) are powerful tools to unravel genomic loci associated with common traits and complex human disease. However, GWAS only rarely reveal information on the exact genetic elements and pathogenic events underlying an association. In order to extract functional information from genomic data, strategies for systematic follow-up studies on a phenotypic level are required. Here we address these limitations by applying RNA interference (RNAi) to analyze 133 candidate genes within 56 loci identified by GWAS as associated with blood lipid levels, coronary artery disease, and/or myocardial infarction for a function in regulating cholesterol levels in cells. Knockdown of a surprisingly high number (41%) of trait-associated genes affected low-density lipoprotein (LDL) internalization and/or cellular levels of free cholesterol. Our data further show that individual GWAS loci may contain more than one gene with cholesterol-regulatory functions. Using a set of secondary assays we demonstrate for a number of genes without previously known lipid-regulatory roles (e.g. CXCL12, FAM174A, PAFAH1B1, SEZ6L, TBL2, WDR12) that knockdown correlates with altered LDL–receptor levels and/or that overexpression as GFP–tagged fusion proteins inversely modifies cellular cholesterol levels. By providing strong evidence for disease-relevant functions of lipid trait-associated genes, our study demonstrates that quantitative, cell-based RNAi is a scalable strategy for a systematic, unbiased detection of functional effectors within GWAS loci.

## Author Summary

Complex traits and diseases are assumed to result from interactions between multiple genes in relevant biological processes. Recent genome-wide association studies have uncovered many novel genomic loci where genes with functional significance are expected. However, functional validation of such genes has thus far remained confined to single gene approaches. Here, we use RNA interference and high-content screening microscopy to profile 133 genes at 56 loci associated with blood lipid traits, cardiovascular disease, and/or myocardial infarction for a function in regulating cellular free cholesterol levels and the efficiency of low-density lipoprotein uptake. Our results suggest that a high number of trait-associated genes have conserved cholesterol-regulatory functions in cells, with several GWAS loci harboring more than one gene of likely functional significance. For a number of genes without previously known lipid-regulatory functions, consequences upon siRNA knockdown positively correlated with cellular levels of LDL receptor, a major determinant of blood LDL levels. Moreover, GFP–tagged fusion proteins of several candidates shifted cellular cholesterol levels to inverse directions than knockdown, and subcellular localization of some candidates was sterol-dependent. Our study generates a valuable resource for prioritization of lipid-trait/CAD/MI-associated genes for future in-depth mechanistic analyses and introduces cell-based RNAi as a scalable and unbiased tool for functional follow-up of GWAS loci.

## Introduction

To date more than 120 genomic loci have been tightly linked to variation in blood lipid levels (low-density lipoprotein (LDL), high-density lipoprotein (HDL), total cholesterol (TC), triglycerides (TG)), susceptibility to coronary artery disease (CAD) and/or myocardial infarction (MI) in more than 23 published large-scale GWAS [1]–[23]. These loci contain 15 out of 18 genes in which variants cause monogenic lipid disorders and further genes with previously defined roles in lipid metabolism [17], supporting the assumption that GWAS enrich for genes with a functional importance on the associated trait [17], [24]–[26]. For the majority of associated loci, however, genes with a function in regulating blood lipid levels or with relevance to CAD/MI have yet to be identified.

Several recent examples show that non-coding variants within associated loci affect the expression of nearby genes, suggesting that cis-regulatory effects on functionally relevant proteins constitute a major trait determinant [17], [24]–[28]. On the level of gene transcripts, such dominant-negative regulatory effects can be closely mimicked by RNAi. RNAi also permits to evaluate the functional consequences of gene knockdown *in vitro* and *in vivo* [29] and has previously enabled us to unravel regulators of cholesterol metabolism from a subset of sterol-regulated genes [30]. This was performed using a strategy that relies on the knockdown of candidate genes in tissue culture cells using siRNA-arrays [30]–[32] and the quantification of how this impacts on two major determinants of blood lipid levels: cellular levels of free cholesterol (FC) and the efficiency of LDL-uptake into cells [30].

## Results/Discussion

**Figure 1:**
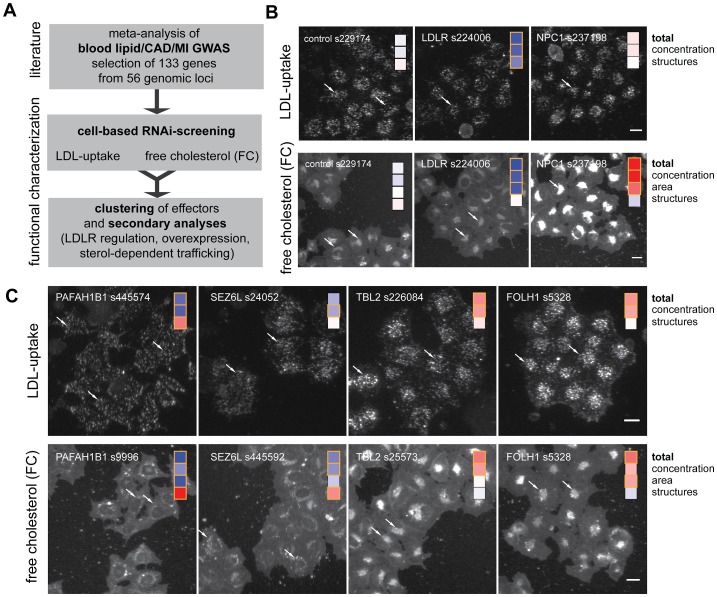
Functional profiling of lipid-trait/CAD/MI associated genes by cell-based RNAi. (A) Workflow of this study. (B,C) Profiling of lipid-trait associated genes for a cholesterol-regulating function in cells was performed by monitoring LDL-uptake (upper panels) and free perinuclear cholesterol (FC; lower panels) in siRNA-knockdown cells (for details, see [30]). Shown are automatically acquired images of Hela-Kyoto cells cultured and reverse siRNA transfected on cell microarrays for 48 h with control siRNAs (B) or indicated siRNAs targeting selected candidate genes increasing (red) or decreasing (blue) typical cellular phenotypes (C; see Figure 2 and Materials and Methods for details). Arrows denote selected compartments representative for respective heatmaps (see text). Bars = 20 µm.

Here we applied this technology with the aim to identify candidate genes within trait-associated loci with a conserved lipid-regulatory function in cells. For this, we functionally analyzed 56 of the 64 genomic loci that were reported until 2009 as associated with lipid traits and/or CAD/MI for genes with a role in cellular cholesterol homeostasis (Figure 1A; Table S1; see Materials and Methods for details). For 38 of the 56 loci all protein-coding genes within ±50 kb of the respective lead SNPs were analyzed, with up to 16 genes at the 19p12 locus. The 18 remaining loci were represented by candidate genes close to the lead SNPs (Table S2). We followed a two-step screening-approach: First, a core gene set of 109 genes was analyzed (“GWAS1”). Promising loci from this gene set were then complemented by additional genes and experimentally re-evaluated (“GWAS2”) (see Materials and Methods). In total, we profiled 133 candidate genes out of which 93 genes had not previously been functionally linked to lipid metabolism (Table S3). Each gene was profiled with 3–5 independent siRNAs, resulting in a total of 534 gene-specific siRNAs tested (Table S4). Uptake of fluorescently-labeled LDL and free perinuclear cholesterol (FC) within siRNA-transfected cells was determined using high-content automated microscopy as described [30] (see Figure S1 and Materials and Methods for how specificity of filipin to reliably detect free cholesterol was assured). For siRNAs analyzed in both, GWAS1 and GWAS2 screens (n = 86), findings correlated well (e.g., Pearson's correlations for the parameter “total cellular intensity” were 0.81 for DiI-LDL uptake and 0.71 for FC) (Table S5), proposing that the results obtained are reproducible and specific. SiRNA-mediated knockdown of multiple known and novel regulators resulted in a consistent increase or reduction of LDL-uptake, FC, or an altered distribution of relevant sub-cellular organelles as signs of perturbed cellular lipid homeostasis (Figure 1B, 1C).

**Figure 2:**
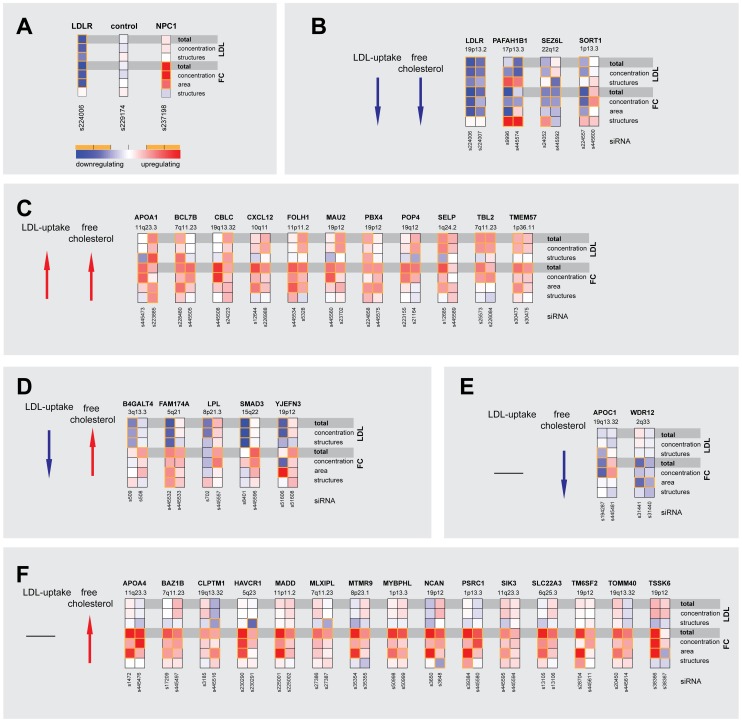
Multiparametric analysis and clustering of functional effector genes. (A) Functional consequences upon knockdown of each candidate gene (using 3–5 different siRNAs/gene) were quantified from microscopic images with regard to seven phenotypic parameters: total cellular LDL-signal; LDL concentration and number of cellular structures; total free cholesterol (FC) signal; and FC concentration, area and number of cellular structures. Shown are heatmaps for 37 out of 55 most pronounced functional effector genes that according to parameter “total cellular intensity” (“total”) of the two strongest effector siRNAs/gene were clustered into five distinct functional groups (B–F) (see Figure S2 and Table S4 for comprehensive datasets). Phenotypes (red, increasing; blue, decreasing) meeting statistical criteria as described in Materials and Methods are framed in orange.

For an objective and quantitative evaluation of our results we developed an automated pipeline for multi-parametric image analysis (Figure S2 and Materials and Methods). From each cell, three (for LDL-uptake) or four (for FC) parameters were measured per siRNA-transfected cell and scored according to effect size (Figure 2A, Figure S3, Table S4 and Materials and Methods). Knockdown of 55 (41%) of the 133 candidate genes tested significantly affected the parameter “total cellular intensity” with two independent siRNAs in at least one of the two screening assays, suggesting these genes as functional effectors on cellular LDL-uptake, FC or both (Table 1, Table S4). This suggested an unexpected high number of effectors, as the typical hit rate of most reported siRNA-screens with unbiased gene sets ranges from 1–6% [32]–[36]. Even in our recent siRNA-screen on a gene set enriched for sterol-regulated genes and known lipid regulators using the same assays as applied here only 25% of the genes scored as effectors [30]. However, of the 63 siRNAs that scored as effectors in GWAS1 and were re-analyzed in validation screens, 30 siRNAs met our stringent statistical criteria also in GWAS2, resulting in a validation rate of 48% (Table S4). Our findings thus strongly support the hypothesis that GWAS enrich for functional regulators of the underlying trait or pathogenic process. They further support previous assumptions that a large proportion of the genes uncovered by GWAS also have a conserved role in tissue culture cells [17], [24], [26].

**Table 1:** Strongest functional effectors in RNAi screens.

		GWAS-literature‡		functional characterization†				database annotation#
GeneName	locus	associated traits*	No. GWAS replicating locus	total LDL-uptake/total free cholesterol (FC)			independent siRNA above controls validating effect	
				siRNA	effect [dev]	*FDR*		described molecular function
**decreased LDL-uptake**								
**LDLR**	19p13.2	LDL, TC, CAD, MI	12	s224006	−1.47	0.001	s224007, s6	LDL receptor
**PAFAH1B1**	17p13.3	HDL	1	s445574	−1.20	0.004	s9998	involved in actin polymerization and dynein-dependent processes (Golgi integrity)
**increased LDL-uptake**								
**TBL2**	7q11.23	HDL, TG	9	s226084	1.32	0.03	s25573	not determined - putative transducer
**FOLH1**	11p11.2	HDL	2	s5328	1.28	0.05	s5327	enzyme, folate hydrolase and carboxypeptidase
**MAU2**	19p12	LDL, TG, TC, CAD	6	s225955	1.20	0.02	s23702	putative role in cohesin complex - chromatin binding
**CBLC**	19q13.32	LDL, HDL, TG, TC, CAD	13	s24224	1.15	0.02	s24223	Regulator of EGFR mediated signal transduction
**decreased free cholesterol**								
**LDLR**	19p13.2	LDL, TC, CAD, MI	12	s224006	−4.11	0.06	s224007, s6	LDL receptor
**PAFAH1B1**	17p13.3	HDL	1	s9996	−3.57	0.08	s9997	involved in actin polymerization and dynein-dependent processes (Golgi integrity)
**SEZ6L**	22q12	CAD	1	s24052	−1.80	0.14	s445592	not determined - putative endoplasmic reticulum function in neurons
**increased free cholesterol**								
**MADD**	11p11.2	HDL	2	s225001	5.53	0.12	s225002	adaptor protein, propagating apoptosis signal by MAPK activation
**PSRC1**	1p13.3	LDL, TC, CAD, MI	13	s39384	4.95	0.10	s445580, s39383	nothing known - putative regulation by p53
**PBX4**	19p12	LDL, TG, TC, CAD	6	s224858	4.94	0.14	s445576	nothing known
**NCAN**	19p12	LDL, TG, TC, CAD	6	s3650	4.74	0.06	s3648, s445568	chondroitin sulfate proteoglycan
**APOA4**	11q23.3	LDL, HDL, TG, TC	12	s1472	4.67	0.11	s445476, s445475	apolipoprotein, HDL
**SLC22A3**	6q25.3	LDL, HDL, TC, CAD	3	s13105	4.23	0.04	s13106	putative potential-dependent cation transporter
**BCL7B**	7q11.23	HDL, TG	9	s228480	4.16	0.18	s445505, s17733	putative role in lung tumor development
**MTMR9**	8p23.1	TG	1	s35354	4.14	0.10	s35355	putative dephosphorylating PI3P protein
**MYBPHL**	1p13.3	LDL, TC, CAD, MI	13	s50998	3.74	0.13	s50999	nothing known
**TM6SF2**	19p12	LDL, TG, TC, CAD	6	s28704	3.71	0.12	s445611	nothing known

‡ GWAS listed in Table S1 and S2.

* blood levels of LDL-C, low-density lipoprotein cholesterol; HDL-C, high-density lipoprotein cholesterol; TG, triglycerides; TC, total cholesterol; CAD, coronary artery disease; MI, myocardial infarction.

† Strongest effector siRNAs (upregulators, red; downregulators, blue) in the two functional assays analyzed. For complete results, see Table S4.

# adapted from www.genecards.org.

55 genes that scored as the most pronounced functional effectors on total LDL-signal and/or cellular FC-levels were selected for further analyses (Tables S3, S6, S7, S8). According to phenotypic fingerprints of the two strongest effector siRNAs/gene (Figure S3 and Materials and Methods), 37 of these genes were tentatively clustered into five distinct functional groups (Figure 2). For 15 genes, direction of functional effects in both screening assays positively correlated (Figure 2B, 2C). Knockdown of 17 genes consistently impacted on FC without obvious effects on LDL-uptake (Figure 2E, 2F), while for 5 genes effects on FC were inversely directed to those observed for LDL-uptake (Figure 2D). For several known effectors, our results were consistent with *a priori* knowledge on the respective genes. For instance, it was recently elegantly demonstrated that altered expression of *SORT1* at the 1p13.3 locus inversely correlates with serum LDL [24]. Consistently, one siRNA targeting *SORT1* induced a strong reduction in FC and also tended to inhibit LDL-uptake, thereby corroborating further that *SORT1* is a key-player in cellular cholesterol homeostasis [24]. Several examples demonstrate that this is most certainly true also for other genes among our effectors that had not previously been linked to lipid metabolism. For instance, two GWAS report association of the *WDR12* locus (2q33) with CAD/MI [10], [16], while a demonstration that this locus is associated with lipid traits is so far missing. However, siRNAs targeting this gene consistently reduced FC, making a lipid-regulatory role for *WDR12* highly likely.

Correlation analysis of the multi-parametric datasets enabled us to hypothesize by which mechanisms some of the previously uncharacterized effectors could possibly impact on cellular lipid homeostasis (Figure 2, Table S5 and Materials and Methods). For instance, a higher number of LDL-positive endosomes and a scattering of FC-retaining organelles upon knockdown of *PAFAH1B1* (Figure 1C) is consistent with a role for this gene in the organization of endosomal membranes [37] and may be a sign of impaired LDL-internalization and/or transport within the endo-/lysosomal system. Effectors such as *TBL2* on the other hand are likely to exert more direct lipid-regulatory functions as knockdown of this gene increased LDL-concentration within endosomes and FC-load, but subcellular structures remained largely unaffected (Figure 1C).

**Figure 3:**
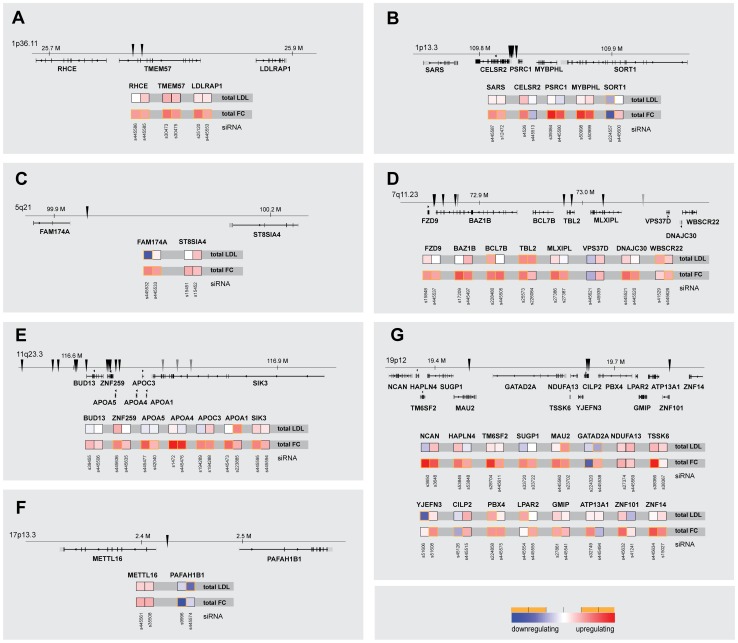
Comparison of multiparametric datasets for neighboring genes within lipid-trait-associated loci. Shown are parameters “total cellular intensity” (“total”) of the two strongest effector siRNAs/gene and relative genomic position of lead SNPs (arrowheads) for seven (A–G) selected lipid-trait/CAD/MI loci in which multiple neighboring candidate genes (±50 kB up-/downstream of lead SNP) were functionally analyzed (see Figure S2 and Table S4 for comprehensive datasets). Phenotypes (red, increasing; blue, decreasing) meeting statistical criteria as described in Materials and Methods are framed in orange.

The identification of genes with relevance for lipid traits and/or CAD/MI from GWAS is complicated by the fact that many lead SNPs locate to gene rich regions [7], [24]. We therefore assessed whether for selected GWAS loci our unbiased approach could help prioritizing functional effectors among several possible candidate genes in such loci. Indeed, in six of the 30 loci for which more than one candidate gene/locus was functionally analyzed, our results suggested one prominent effector gene. Most surprisingly, in 9 of these 30 loci knockdown of more than one gene per locus affected cellular cholesterol homeostasis (Figure 3). For instance, of the 8 genes analyzed at the 7q11.23 locus (Figure 3D) not only *MLXIPL* as the most likely candidate to explain association with TG [8], but also five other genes scored as significantly increasing FC, among them *TBL2*, knockdown of which also induced the strongest observed stimulation of LDL-uptake. Similar observations for novel effectors in addition to genes with well-characterized lipid-regulatory functions were made for loci 1p36.11, 11q23.3 or 12q24.11 among others (Figure 3). Furthermore, while none of the 16 candidate genes at the 19p12 locus was previously ascribed a lipid-regulatory function, ten scored as effectors with two independent siRNAs in at least one of the two functional assays, which might reflect the substantial pleiotropy at this locus in six different GWAS [1], [6], [7], [17], [20], [21] (Table S1).

**Figure 4:**
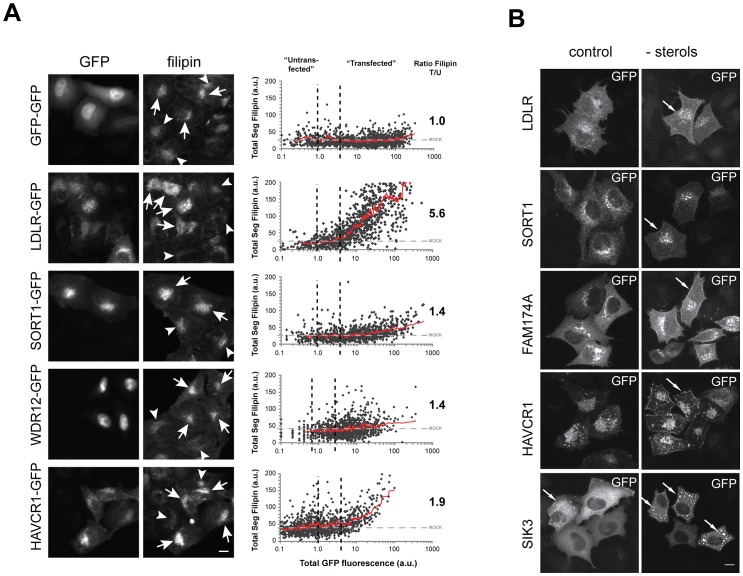
Impact on FC levels and subcellular localization of GFP–tagged candidate genes. (A) cDNAs encoding for indicated candidate genes linked to GFP were transiently expressed in Hela-Kyoto cells and impact on cellular FC levels was analyzed (see Materials and Methods, Figure S6 and Table S8 for comprehensive datasets). Arrows denote “transfected”, arrowheads “untransfected” cells. See Materials and Methods for definition of threshholds (dashed lines in graphs).Graphs depict total segmental filipin signal plotted against total cellular intensities in the GFP-channel. Each dot reflects one individual cell, trend lines are given in red. Numbers indicate mean ratios of FC in GFP-positive relative to non-expressing cells within the identical dish (n = 3–4 experiments). (B) Maximal projections of confocal stacks showing representative GFP-cDNA expressing cells under control and sterol-depleted conditions (see Materials and Methods). Arrows denote cellular compartments with increased signals upon sterol-depletion. Bars = 10 µm.

In order to gain initial insight into the mechanisms how newly identified effectors could functionally contribute to cholesterol homeostasis, we validated data from our RNAi-screens with a set of secondary assays (Table 2; Figure 4; Figures S4, S5, S6, S7; Tables S6, S7, S8; and Materials and Methods). For instance, an enzymatic assay (see Materials and Methods) applied under screening conditions showed a considerably lower dynamic range to detect changes in cellular cholesterol levels than our image-based approach using filipin. However, results from both approaches correlated well (R^2^ = 0.48, p<10^−6^), and biochemical analyses corroborated six candidate genes (*C12orf43*, *GATAD2A*, *SEZ6L*, *SORT1, TOMM40*, *TSSK6*) as cholesterol regulators. Likewise, 18 out of 70 effector-siRNAs tested (26%) were also above thresholds when FC was measured from HuH7 liver cells and two candidate genes (*BAZ1B*, *HAVCR1*) could be validated with two independent siRNAs also in this cell model (Figure S4, Table S7). Interestingly, despite similar knockdown efficiencies at the protein level (Figure S4C), phenotypic changes upon knockdown of individual effectors were in general less pronounced in HuH7 compared to Hela cells (Figure S4D). One explanation why only a low number of candidate genes could be confirmed in HuH7 cells could be a reduced sensitivity of the filipin assay to monitor changes in free cholesterol in this cell line. Alternatively, liver cells could have mechanisms that compensate in parts the knockdown of specific candidate genes tested that are absent or less effective in Hela cells. One way to identify such compensatory mechanisms in the future could be double knockdown experiments where the candidate genes identified in this work in Hela cells would be knocked-down in combination with putative genes accounting for the compensatory mechanisms in HuH7 cells. We further assessed whether knockdown of effector genes affected mRNA and protein levels of LDLR, a major determinant of blood LDL-levels [38]. Remarkably, for 19 out of 35 effector genes tested at least one siRNA also affected LDLR expression, either on the mRNA level (3 siRNAs), protein level (15 siRNAs) or both (*CETP*). In several instances LDLR levels and phenotypic effects on LDL-uptake and/or FC positively correlated. For instance, impaired LDL-uptake upon knockdown of *B4GALT4* and *PAFAH1B1* could be directly caused by a lack of LDLR, as siRNAs targeting these genes also reduced LDLR-protein levels. Perturbed levels of LDLR-mRNA or protein on the other hand are consistent with increased or decreased FC upon knockdown of *CXCL12*, *TSSK6* or *WDR12*. These results support a role for these effectors on LDLR and suggest a mechanism how variants affecting expression and/or function of these genes may impact on lipid traits and/or CAD/MI risk. No direct correlation between LDLR levels and cellular phenotype was observed for most other effectors. Such results may be explained by compensatory cellular mechanisms that tightly control LDLR at the transcriptional and post-transcriptional level [39], [40]. Alternatively, they could as well hint at yet unknown mechanisms and pathways that contribute to control blood lipid levels independent of LDLR, which await clarification in further studies.

**Table 2:** Selected results of secondary assays.*

	Screen		FC validation		LDLR		Overexpression		
Gene	LDL-uptake	FC	cellular cholesterol (enzymatic)	FC in liver-derived cell line (HuH7)	mRNA	protein	FC	sterol dependent relocalization	most prominent subcellular localization
**BAZ1B**	n.s.	**+**	n.s.	**+**	n.s.	n.s.	**+**	no	nuclear
**CLPTM1**	**(+)**	**+**	n.s.	n.s.	n.d.	n.d.	n.s.	no	perinuclear structures
**CXCL12**	**(+)**	**+**	n.s.	n.s.	n.s.	**(+)**	**−**	no	perinuclear structures
**FAM174A**	**(−)**	**+**	n.s.	n.s.	n.s.	**(−)**	**+**	yes	perinuclear structures
**FOLH1**	**+**	**+**	n.s.	**(+)**	n.s.	n.s.	**+**	no	perinuclear structures
**HAVCR1**	n.s.	**+**	n.s.	**+**	n.d.	n.d.	**+**	yes	perinuclear structures
**LDLR**	**−**	**−**	**(−)**	n.s.	**(−)**	**(−)**	**+**	yes	perinuclear structures
**MAU2**	**+**	**+**	n.s.	n.s.	n.s.	n.s.	**+**	no	nucleus, cytoplasm
**MLXIPL**	n.s.	**+**	n.s.	**(+)**	n.s.	**(+)**	n.s.	no	cytoplasmic reticular
**MYBPHL**	**(+)**	**+**	n.d.	n.s.	n.d.	n.d.	**+**	no	nucleus, cytoplasm
**NCAN**	**(+)**	**+**	n.s.	**(+)**	n.d.	n.d.	**+**	no	perinuclear structures
**PAFAH1B1**	**−**	**−**	n.s.	**(−)**	n.s.	**(−)**	n.s.	no	cytoplasmic reticular
**POP4**	n.s.	**+**	n.d.	−/+	n.s.	**(−)**	n.s.	no	nucleus, nucleolus
**PSRC1**	n.s.	**+**	n.s.	n.s.	n.d.	n.d.	n.s.	no	cytoplasmic reticular
**SEZ6L**	**(−)**	**−**	**(−)**	n.s.	n.s.	**(+)**	**+**	yes	perinuclear structures
**SMAD3**	**(−)**	**+**	n.d.	**(+)**	n.s.	n.s.	**+**	no	nucleus
**TBL2**	**+**	**(+)**	n.s.	**(+)**	n.s.	**(+)**	n.s.	no	perinuclear structures
**TM6SF2**	**(−)**	**+**	n.d.	**(+)**	n.s.	**(−)**	n.s.	no	perinuclear structures
**TMEM57**	**(+)**	**+**	n.s.	**(+)**	n.s.	n.s.	**+**	no	reticular
**TOMM40**	**(−)**	**+**	**(−)**	n.s.	n.d.	n.d.	n.s.	no	reticular
**YJEFN3**	**(−)**	**+**	n.d.	n.s.	n.s.	**(−)**	n.s.	no	nucleus, cytoplasm
**APOA1**	**(+)**	**(+)**	n.s.	n.s.	n.s.	**(−)**	n.s.	no	perinuclear structures
**APOC1**	**(+)**	(−/+)	n.s.	n.s.	n.d.	n.d.	n.s.	no	perinuclear structures
**B4GALT4**	**(−)**	**(+)**	n.d.	n.s.	n.s.	**(−)**	n.s.	no	perinuclear structures
**LPL**	**(−)**	**(+)**	n.d.	n.s.	n.s.	n.s.	n.s.	no	perinuclear structures
**SIK3**	n.s.	**(+)**	n.d.	n.s.	n.s.	n.s.	n.s.	yes	cytoplasm, punctuate structures
**SORT1**	**(−)**	**(−)**	**(−)**	n.s.	n.s.	n.s.	**+**	yes	perinuclear structures
**TSSK6**	n.s.	**(+)**	**(+)**	**(+)**	**(+)**	n.s.	n.s.	no	nucleus
**WDR12**	n.s.	**(−)**	n.s.	n.s.	**(−)**	n.s.	**+**	no	nucleoli

* Summarized results from secondary assays for 29 exemplary effector genes. For full datasets, see Table S7. For RNAi-screens, + (increase) and − (decrease) denote genes validated by two independent siRNAs, (+) and (−) genes scoring with 1 siRNA, and (−/+) genes where one siRNA scored in opposite directions each. n.s., not significant; n.d., not determined.

29 promising effector genes were further followed up in systematic overexpression experiments. For this, GFP-tagged proteins were transiently transfected into Hela cells and cellular FC levels upon candidate gene overexpression were quantified (see Materials and Methods). Under our experimental settings, overexpression of 14 of the 29 candidates tested significantly increased or reduced FC levels (Figure 4A, Table 2, Table S8). For four candidates (*CXCL12*, *SEZ6L*, *SORT1*, *WDR12*) overexpression and knockdown shifted FC levels into opposite directions, thereby confirming levels of these genes as critical for maintenance of cellular cholesterol homeostasis. It is highly likely that future, more tailored studies will corroborate overexpression also of further candidates from our gene set as regulating cellular cholesterol that were here missed due to the screening format chosen. For instance, overexpression of CXCL12, a secretory cytokine highly associated with CAD/MI [41], significantly reduced FC (as opposed to increased FC and LDL-uptake in CXCL12 knockdown cells) in the whole cell population, but not individual GFP-expressing cells, indicating that CXCL12 might exert its cholesterol-modulating functions *in trans*, via being secreted (Table S8). Interestingly, candidates such as BAZ1B or HAVCR1 strongly increased FC in a concentration-dependent manner, similar to the effect obtained by knockdown of these genes. One explanation for these apparently contradictory results could be that such genes achieve their functions by interplay with other factors in known or yet unknown lipid-regulatory pathways. Under that scenario, knockdown of individual candidates would also affect the function of their interactors and thereby result in complex phenotypic readouts. Likewise, elevated levels of GFP-tagged versions of these genes could affect complex formation and function (e.g. due to the GFP-tagging of the proteins) and thus result in similar effects on cholesterol homeostasis as observed by their knockdown. Further work will be necessary to identify the cellular mechanisms to support these hypotheses. Confocal imaging revealed that many of the GFP-tagged proteins analyzed here localize to subcellular organelles of known relevance to cellular lipid homeostasis (Figure S6). Interestingly, however, several effectors were enriched in organelles without previous lipid-relevant functions, e.g. nucleoli (*WDR12*). Moreover, for six candidates (*FAM174A*, *HAVCR1*, *LDLR*, *SEZ6L*, *SIK3*, *SORT1*) we observed a re-localization to alternative organelles when cholesterol-levels were reduced (Figure 4B), which is a feature also of other crucial regulators of cellular cholesterol homeostasis [30], [41].

Taken together, our study demonstrates the potency of RNAi combined with systematic follow-up analyses to identify and profile functionally relevant effector genes within GWAS loci in an objective and unbiased manner. Several independent studies on individual candidate genes are well in line with some of the findings described here. For instance, it was recently shown that overexpression and knockdown of TBL2 inversely modulates cellular cholesterol in HEK293 and bladder cancer cells [42]. Likewise, SIK3-deficient mice have low levels of serum HDL and total cholesterol, but under a lipid-rich diet cholesterol accumulates in mouse livers [43]. Although systematic studies in mammalian cells have contributed significantly to our understanding of human lipid biology and disease [44], future work using e.g. suitable animal models will be necessary to test the in vivo roles in cholesterol metabolism of the candidate genes identified in the cell-based work here. As cell-based RNAi is scalable up to the whole genome [32], [45], its potential to complement genomic data with information on the functional significance of trait-associated candidate genes and sequence variants is considerable. Together with functional studies in animal models and more thorough approaches for cellular and biochemical profiling of candidates as we have initiated here, systematic cell-based functional analyses may thus emerge as a key technology for the detection of genes and pathways underlying a biological trait or disease process.

## Materials and Methods

### Cells and reagents

Hela-Kyoto cells are a strongly adherent Hela isolate (gift from S. Narumiya, Kyoto University, Japan) also used in previous siRNA-screens [30], [32]. HuH7 cells were obtained from the Japanese Collection of Research Bioresources. Filipin III (Sigma) was prepared as a 1 mg/ml stock-solution in di-methyl-formamide. DRAQ5 (Biostatus), ER-tracker Blue/White DPX (Molecular Probes), Fugene 6 (Roche). Lipofectamine 2000 (Invitrogen), 2-hydroxy-propyl-beta-cyclodextrin (HPCD) (Sigma), DiI-LDL (Inivtrogen) and Benzonase (Novagen) were purchased from the respective suppliers.

### RNAi screening

#### SiRNA selection, production of transfected siRNA microarrays, and functional assays

56 of the 64 genomic loci that until 2009 were reported as associated with lipid traits and/or CAD/MI in 23 large-scale GWAS 1–7,9–23,30 were considered for RNAi-screening (Table S1). Eight published loci were omitted from analysis either due to the absence of annotated genes in the vicinity of the lead SNPs (2q36.3 [14], 4q12 [19], 9p24.3 [19], 15q14 [13]) or because of annotation discrepancies of published genomic positions relative to the current human reference genome GRCh37.p6, (Ensembl 66, February 2012) (1q25.2 [19], 1q43 [22], 9q31.3 [19], 20q13.12 [12]). Out of the 56 selected loci, 40 have since been replicated as highly associated (p<7×10^−6^) in at least a second independent GWAS (Table S1). For 36 loci association with more than one of the six traits analyzed (LDL-C, HDL-C, TC, TG, CAD, MI) was observed (Table S1, Table S2). Based on proximity to the lead SNP given in the literature, 106 candidate genes were selected from the 56 GWAS-loci for two primary RNAi-screens (“GWAS1”). For follow-up screens (“GWAS2”), 17 loci were complemented with 27 additional candidate genes so that for 38 loci all protein-coding genes within ±50 kbp of all reported lead-SNPs were covered. This resulted in a total of 133 genes analyzed in this study (Table S3).

Knockdown of each of the 133 candidate genes was performed in Hela-Kyoto cells cultured at 37°C/5%CO_2_ in DMEM/2 mM L-glutamine(Sigma)/10%FCS(PAA) and transfected with 21 nt SilencerSelect siRNAs from Applied Biosystems for 48 h. For GWAS1, each gene was silenced by three independent pre-designed siRNAs. For candidate genes that were re-analyzed in GWAS2 (n = 76), two siRNAs targeting the coding region were complemented by two additional siRNAs directed against the untranslated regions (UTR) of a respective target-mRNA (Table S4). SiRNAs against the UTR were designed using the BLOCK-iT RNAi Designer tool (https://rnaidesigner.invitrogen.com/rnaiexpress/). All siRNA sequences were mapped to the human reference genome GRCh37 (Ensembl 66, February 2012) using the in-house software tool bluegecko (J.K. Hériché, unpublished). This provided information on how many protein-coding transcripts per gene were targeted by an individual siRNA (Table S4) and allowed us to identify unspecific siRNAs that targeted also alternative human mRNAs, showed a mismatch to the reference sequence of the respective target gene or were directed against transcripts not anymore considered as protein coding. Based on these results, 24 siRNAs were thus excluded from further analysis (including all siRNAs targeting *APOC2*, *SLC35G5* and *LPAL2*; Table S4).

Glass-bottomed chambered cell culture slides and 384-well plates coated with siRNAs for solid phase reverse-transfection of cells were produced as described [31], [46]. Of the 384 positions, 16 contained non-silencing control siRNA (s229174), three (GWAS1) or eight (GWAS2) positions, respectively, transfection mix without siRNA (mock), four positions INCENP-siRNA (to control for transfection efficiency [32]), eight positions siRNAs targeting LDLR (s237197 in GWAS1, s224006 in GWAS2) as a positive control for LDL-uptake, and eight positions siRNAs targeting NPC1 (s237198) as a positive control for free cholesterol (FC). Assays to monitor cellular LDL-uptake and FC as well as image acquisition were performed as described using an Olympus IX81 automated microscope and an UPlanSApo 10×/NA 0.40 objective [30]. FC was analyzed from 384-well plates in three (GWAS1) or four (GWAS2) biological replicas with four images per well. For this, 48 h before analysis 900 Hela-Kyoto cells/siRNA-coated 384-well were seeded using a cell seeder. HuH7-cells were seeded at a density of 500 cells/siRNA-coated 384-well and cultivated for 72 hrs before analysis. LDL-uptake was analyzed from cell arrays with ten (GWAS1) or eight (GWAS2) biological replicas by seeding 10^5^ Hela-Kyoto cells per array 48 h before analysis.

#### Analysis of RNAi screens

All 21,494 images from GWAS1 and GWAS2 were visually quality controlled in order to exclude out of focus and otherwise not analyzable images (e.g. due to aberrant cell density or dust particles). This resulted in 20,078 images (93%) for further analysis comprising a total of ∼2.5×10^6^ cells with a median of 835 cells per siRNA for LDL-uptake and of 2778 cells per siRNA for FC.

Automated image analysis was performed using the open source software Cellprofiler (http://www.cellprofiler.org [47]). In brief, areas of single cells were approximated by stepwise dilation of masks generated from images of DRAQ5-stained cell nuclei (for FC) or propagating the nuclear masks on the dpx (GWAS1) or DRAQ5 (GWAS2) channels (for LDL-uptake). Using a project-specific module (MorphoQuant), filipin (for FC) or DiI-signal (for LDL-uptake) was quantified from masks representing intracellular areas that were determined by local adaptive thresholding according to pre-defined parameters for size and shape (Figure S1). From each cell, three (for LDL-uptake) or four (for FC) parameters were quantified reflecting (i.) total signal intensity above local background within these masks/cell (“total”), (ii.) mean signal intensity above local background within these masks per cell (“concentration”), (iii.) number of masks per cell (“structures”) and (iv.) total area covered by masks per cell (“area”) for FC. Means were calculated from all cells per image and different images from the same biological replicate were averaged. Then, for each siRNA/biological replicate a “deviation value” was calculated (deviation_replicate,siRNA_) by subtracting from the mean signal of a respective siRNA the mean signal of the 16 negative control siRNAs of the identical replica and division by twice the error of these controls (1,2). With this, the “deviation value” is calculated as a z-score, but instead of the standard deviation twice the absolute error was used, which takes into account variation in signal intensities. This allowed for normalization and taking into account the variation of negative controls. Deviation values of the negative controls were controlled for normal distribution by qqplot and Shapiro-Wilk test for each parameter. To antagonize plate effects on the 384-well plates, parameters for siRNAs localizing to wells at the edge of the plate (region 1), those neighboring these wells (region 2) and the rest of the plate (region 3) were normalized to controls within the respective region. To define the functional effect of a respective siRNA from 3 to 10 biological replicas, deviation values from all biological replicas were averaged (3).
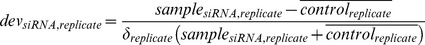
(1)

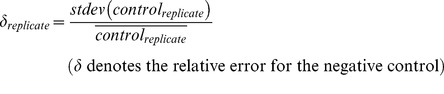
(2)

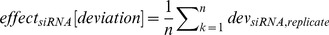
(3)SiRNAs were defined as effectors in an assay when the mean deviation value for a respective siRNA on the parameter “total signal intensity within masks/cell” (“total”) was above or below the mean of any of the negative controls (comprising 16 negative control siRNAs and three to nine mock transfection controls) ± 3-times the standard error of the mean (the standard deviation of all negative control values divided by the square root of the number of biological replicates). Identical criteria were applied to identify siRNAs exceeding thresholds (framed orange in Figures and colored in Table S4) for the other parameters analyzed. When treating the negative controls as candidate siRNAs, none of the up to 16 control siRNAs or three (GWAS1) to nine (GWAS2) mock controls passed these thresholds in none of the screens. A gene was considered a hit if these thresholds were exceeded by two independent siRNAs.

To test for a possible interdependence of the parameters measured, pairwise Pearson's correlation values between the seven listed plus two additional parameters/assay (reflecting number and area of cells) were calculated across the entire dataset (comprising 557 independent siRNAs) (Table S5). For instance, in both assays the parameters reflecting “total” and “concentration” positively correlated. This is well in line with observations for epidermal-growth factor (EGF) and transferrin (TF) endocytosis [45] and corroborates that the uptake of LDL into cells underlies similar basic mechanisms as do alternative endocytic markers [38]. The parameter “total” did not strongly correlate with with number of Cells (nCells) or cell area (AreaCell), indicating its robustness to variation in cell density and size. Conversely, all seven parameters used for quantifications strongly correlated between GWAS1 and GWAS2-screens, reflecting a high reproducibility of individual results.

### Secondary analyses

#### Quantification of cellular cholesterol

Filipin was recently reported to under certain conditions also bind to GM1 as well as further membrane lipids [48]. In order to assure specificity of filipin to accurately reflect free cholesterol under the experimental settings used here (Figure S1), SphingoStrips (Echelon Biosciences) and MembraneLipidStrips (Echelon Biosciences) representing 26 different lipids were washed for 5 min in PBS before staining for 30 min with 50 µg/ul filipin III in PBS. Then, strips were washed three times with PBS and illuminated with UV-light for readout. Cholesterol was the only lipid detected under these conditions, demonstrating the high specificity of filipin III. Moreover, addition of 1 µM GM1 for 15 min to the medium of Hela-Kyoto cells cultured in 6-well dishes prior to fixation doubled the levels of cell-associated GM1 (as detected by the GM1-stain cholera toxin AF-568), but did not affect total perinuclear or cell surface-associated filipin signals. Conversely, sterol-depletion by culture in DMEM/2 mM L-glutamine(Sigma)/0.5%BSA for 16 h followed by exposure for 45 min to 1% (w/v) HPCD [30] strongly reduced filipin intensities without changing cellular cholera toxin signals. These results suggested that a possible cross-reactivity of filipin with GM1 or other abundant membrane lipids is negligible under our experimental settings (Figure S1).

For enzymatic determination of cellular cholesterol, the Amplex Red Cholesterol Assay Kit (Life Technologies) was used according to manufacturer's instructions. Hela-Kyoto cells were seeded at a density of 3000 cells/well on 96-well plates (Nunc) coated with siRNAs as described above. 48 h post transfection, cells were washed three times with room-temperature PBS and 50 µl 1× reaction buffer was added (100 mM potassium phosphate, 50 mM NaCl, 50 mM cholic acid, 0.1% Triton-X-100), followed by 50 µl/well of Amplex Red solution (300 µM Amplex Red, 2 U/ml horse-radish peroxidase, 2 U/ml cholesterol oxidase in 1× reaction buffer). Upon 45 min at 37°C plates were analyzed in a Tecan2 plate reader at excitation wavelengths 535–555 nm and emission wavelengths 590–610 nm. SiRNAs were considered as effectors on cellular cholesterol if means from 3–6 experimental replicas deviated >2 standard deviations from the mean of negative controls (control siRNA and mock) and showed a p<0.05 in two two-sided Student's t-tests against both, control siRNA and mock transfection controls.

#### FC determination from HuH7-cells

FC levels from HuH7 liver cells were determined as described above. However, to accommodate for considerable morphological heterogeneity between the cells of one experiment, quantitation was limited to cells that were visually assessed as correctly segmented. Selection of 150–600 cells/siRNA from three independent biological replicates was assisted by a customized ImageJ plugin (C. Tischer, unpublished). SiRNAs were considered as effectors if ≥50 cells/experiment could be quantified, mean deviation values were outside a pre-defined confidence interval (mean ± 2-times the standard error of the mean of both negative controls) and a two-sided Student's t-test was p<0.05 in at least two biological replicas.

#### Analysis of LDL–receptor regulation

For analysis of LDLR mRNA and protein levels, Hela-Kyoto cells seeded at a density of 3·10^4^ cells/well and cultured in DMEM/2 mM L-glutamine/10% FCS were reverse siRNA-transfected for 48 h on 96-well plates (Nunc). The mRNA was extracted and cDNAs obtained by using the Cells-to-CT kit (Applied Biosystems #4402955). QPCR was performed in triplicates from 3–4 biological replicates/siRNA with the following primers: LDLR fwd 5′-AGTGTGACCGGGAATATGACT-3′ rev 5′-CCGCTGTGACACTTGAACTT-3′; GAPDH fwd 5′-CATGAGAAGTATGACAACAGCCT-3′ rev 5′-AGTCCTTCCACGATACCAAAGT-3′; ACTB fwd 5′-CGCGAGAAGATGACCCAGAT-3′ rev 5′-TCACCGGAGTCCATCACGAT-3′. For each siRNA the fold change (2^−ΔΔCT^) of LDLR-mRNA was normalized to that of *ACTB* and *GAPDH* (Figure S5 and Table S6). SiRNAs were considered as differentially regulating LDLR-mRNA levels if levels deviated >2 standard deviations from the mean of control (untreated, mock) cells (n = 16; STDEV_LDLR/GAPDH_ = 0.21; STDEV_LDLR/ACTB_ = 0.20) and four two-sided Student's t-tests against both untreated and mock transfection controls of both housekeeping genes (*ACTB*, *GAPDH*) resulted in a p<0.01.

For Western Blot, siRNA-transfected cells were lysed in 40 µl SDS-loading buffer and subjected to immunoblotting with α-LDLR (Cayman Chemicals) and α-tubulin (Neomarkers) antibodies. Integrated density of bands was normalized to the average integrated density of bands from untreated and control siRNA treated samples on the identical SDS-gel and absolute LDLR protein signal as well as LDLR normalized to tubulin (LDLR/tub) was measured (Figure S6 and Table S6). SiRNAs were considered as differentially regulating LDLR-protein levels if levels deviated >2 standard deviations from the mean of control (untreated, control siRNA) cells (n = 34; STDEV_LDLR_ = 0.13; STDEV_LDLR/tub_ = 0.18) and four two-sided Student's t-tests against both untreated and control siRNA treated controls of both normalization controls (LDLR, LDLR/tub) resulted in a p<0.01.

#### GFP–cDNA overexpression and analysis

For 29 candidate genes, sequence-verified human cDNA-clones carboxy-terminally linked to EGFP were obtained (SourceBiosciences) (Table S8). Hela-Kyoto cells were seeded at a density of 8×10^5^ onto glass coverslips in 12-well plates (Nunc), cultured in DMEM/2 mM L-glutamine/10% FCS for 16 h at 37°C/5% CO_2_, and fluid-phase transfected with 2 µg cDNA/well with Lipofectamine2000 (Invitrogen) according to manufacturer's instructions. 24 h post transfection cells were fixed and FC was analyzed as described above using a Cellprofiler pipeline together with a customized ImageJ plugin (C. Tischer, unpublished). Additionally, integrated densities from cell masks in the GFP-channel were recorded. For each construct, ∼500 cells were randomly selected from 3–4 replica experiments and non-GFP expressing (“untransfected”) cells determined as showing GFP-signal intensities <97% of mock transfected cells. Conversely, GFP-cDNA expressing (“transfected”) cells were defined as showing cellular GFP-signals >4-fold above the upper threshold of “untransfected” cells. For each cell, total segmental filipin intensities were determined relative to cellular GFP-levels (Figure 4, graphs). For each candidate gene and the ratio of filipin signal in “transfected” relative to mock-transfected, as well of “transfected” relative to “untransfected” cells in the same dish was calculated as means from all experimental replicas. Effects were considered as significant when a two-tailed Student's t-test resulted in p-values <0.01 in 2 (*) or 3–4 (***) experimental replicas (Table S8).

For determination of subcellular localizations (Figure S6), Hela-Kyoto cells were seeded at a density of 4×10^5^ cells/well onto glass coverslips in 24-well plates (Nunc), cultured in DMEM/2 mM L-glutamine/10% FCS for 16 h at 37°C/5% CO_2_, and fluid-phase transfected with 1 µg cDNA/well Fugene 6 (Roche) or 2 µg cDNA/well Lipofectamine2000 (Invitrogen) according to manufacturer's instructions. For depletion of cellular sterols, medium was exchanged 12 h post transfection for DMEM/2 mM L-glutamine/0.5% lipoprotein-depleted serum (LDS). After 16 h, 1%(w/v) HPCD was added to cells for an additional 3 h as described [30]. Images were acquired on a Zeiss LSM780 confocal microscope using a 63×/NA 1.4 oil objective.

## Supporting Information

Figure S1 Assessing specificity of filipin III to detect free cholesterol. (A) Lipid strips representing 26 different lipid species were incubated for 30 min with 50 µg/ul filipin III in PBS and imaged with UV-light. (B–D) Hela-Kyoto cells were challenged by 15 min addition of 1 µM GM1 ganglioside to the medium or sterol-depletion by culture in DMEM/2 mM L-glutamine/0.5%BSA for 16 h followed by exposure for 45 min to 1% (w/v) hydroxyl-propyl-beta-cyclodextrin (HPCD) (− sterols) [30]. Images of filipin III or cholera toxin-AF568 (CTxB) stained cells were acquired on an automated widefield microscope. Bar = 20 µm. Total integrated intensities in perinuclear areas (filipin) or whole cell masks (CTxB) (C) or in a 100×100 pixel area at the cell peripheries (D) were quantified from 2 experimental replicas. Shown are means ± range of independent experiments.
(PDF)

Figure S2 Pipelines for automated multi-parametric image analysis. Shown are representative images (left panels) acquired by automated fluorescence microscopy during RNAi-screening. (A) For measuring cellular LDL-uptake Hela-cells exposed to fluorescent DiI-LDL for 20 min at 37°C were fixed and stained for DRAQ5 (nuclei) and ER-Tracker dpx (endoplasmic reticulum). (B) For measuring FC, cells were stained with cholesterol-binding dye filipin III and DRAQ5 (for details see [30]). Masks (right panels) for quantification of DiI and filipin signal intensities from areas approximating whole cells and subcellular compartments were generated using Cellprofiler software (for details see Materials and Methods).
(PDF)

Figure S3 Multi-parametric analysis and clustering of 133 genes representing 56 lipid-trait/CAD/MI GWAS-loci. Functional consequences upon knockdown of each candidate gene were quantified from microscopic images with regard to seven phenotypic parameters: total cellular LDL-signal; LDL concentration and number of cellular structures; total free cholesterol (FC) signal; and FC concentration, area and number of cellular structures. For each of the 133 genes analyzed in this study, heatmaps of the two siRNAs causing the strongest effect on the parameters total cellular signal intensities (grey bar) are shown (see Table S4 for numeric data). Phenotypes (red, increasing; blue, decreasing) where mean effect size was above or below that of all 19 non-silencing control siRNAs are framed in orange. Genes are sorted according to chromosomal position.
(PDF)

Figure S4 Analysis of free cholesterol (FC) levels in siRNA-treated HuH7 liver cells. HuH7 liver-derived cells were reverse siRNA-transfected for 72 h with 84 siRNAs targeting 42 selected candidate genes from GWAS-loci. (A) Representative images of cells treated with indicated siRNAs acquired by automated fluorescence microscopy during RNAi-screening. (B) Mean perinuclear total free cholesterol (FC) signals relative to control siRNA-treated cells in Hela-Kyoto (open bars) relative to HuH7 cells (filled bars). SiRNAs where mean functional effects in HuH7 cells exceeded upper or lower thresholds (indicated by orange lines) in ≥2 experimental replicates (by ≥2 standard deviations of negative controls) are highlighted by asterisks (see Table S7 for numeric data). (C,D) Comparison of protein knockdown efficiencies (C) and filipin signals (D) for two selected effector genes (NPC1, PAFAH1B1) in Hela-Kyoto and HuH7 cells. Note that at similar knockdown efficiencies functional effects were considerably less prominent in HuH7 relative to Hela-cells. Bars = 20 µm.
(PDF)

Figure S5 LDLR mRNA levels upon RNAi of 35 lipid-trait/CAD/MI associated genes. LDLR mRNA levels in Hela-Kyoto cells were assessed by qPCR upon knockdown of 105 siRNAs targeting 35 selected candidate genes from GWAS-loci. LDLR levels were normalized to housekeeping genes GAPDH (blue) and ACTB (green). For each siRNA the mean fold change ± SEM of 3–4 biological replicates is shown (see Table S6 for numeric data). Significance thresholds (≥2 standard deviations of negative controls; orange lines) and siRNAs reaching significant p-values in two-sided Student's t-test (*<0.01; **<0.001; ***<0.0001) are indicated.
(PDF)

Figure S6 LDLR protein levels upon RNAi of 35 lipid-trait/CAD/MI associated genes. LDLR protein levels in Hela-Kyoto cells were assessed by Western Blot upon knockdown of 105 siRNAs targeting 35 selected candidate genes from GWAS-loci. LDLR levels quantified from blots were compared either directly or normalized to housekeeping gene α-tubulin. Shown are results from 3 biological replicates (see Table S6 for numeric data). Arrows denote siRNAs that significantly (*<0.01; **<0.001; ***<0.0001) reduced (blue) or increased (red) LDLR protein levels above or below thresholds (≥2 standard deviations of negative controls). Bands from lysates where no reliable results could be obtained were crossed out and excluded from quantitative analysis.
(PDF)

Figure S7 Subcellular localization of 29 GFP–tagged proteins encoding lipid-trait/CAD/MI associated genes. Hela-Kyoto cells were transfected for 24 h with cDNAs expressing indicated proteins carboxy-terminally linked to eGFP. Cells were cultured either under control or sterol-depleted conditions (by culture in DMEM/2 mM L-glutamine/0.5%BSA for 16 h followed by exposure for 3 h to 1% (w/v) hydroxyl-propyl-beta-cyclodextrin (HPCD) (− sterols) [30]. Shown are maximal projections of confocal stacks of representative cells. Proteins for which subcellular localization differed between control and sterol-depleted conditions are highlighted in grey and with arrows. Bar = 10 µm.
(PDF)

Table S1 Overview of genes analyzed and the GWAS that show association to blood lipid levels and/or CAD/MI.
(XLS)

Table S2 Selection of genes based on SNPs from lipid GWAS. Loci and lead-SNPs used to select genes for RNAi screens. GWAS in italics appeared after start of the study. For 38 of the 56 loci all protein-coding genes within ±50 kb of the respective lead SNPs were analyzed (locus in bold). The 18 remaining loci were represented by candidate genes close to the lead SNPs.
(XLS)

Table S3 Genes analyzed in RNAi screens and a priori knowledge on molecular function. Comprehensive GWAS gene set analyzed in this study, listed according to HGNC Symbol and Ensembl Gene ID. It has been looked up whether the genes have been previously linked to lipid metabolism using GO annotation (search terms: cellular lipid metabolism, cellular response to cholesterol, cholesterol*, lipid*, lipoprotein*, triglyceride*, high-density*, low-density*, very-low-density*), whether they have been linked to a monogenic lipid disorder [17], or whether they scored as hits in the indicated previous genome-wide RNAi screens [32], [45], [49]. Gene functions were adapted from www.genecards.org.
(XLS)

Table S4 Results from GWAS RNAi screen. Each gene was targeted by 3–5 different siRNAs which are shown with siRNA ID (Applied Biosystems), sequence and number of targeted out of total protein coding transcripts. SiRNAs targeting untranslated gene regions (UTR) are indicated. For mapping of siRNA sequences to the reference genome, see Materials and Methods. Italics, siRNAs that did not show perfect mapping with reference sequence (asterisks give explanations). Quantiative data for indicated parameters are given as deviation units (see Materials and Methods). SiRNAs with mean total cellular signal intesities (“total”) below or above thresholds (LDL GWAS1 [−0.50, 0.81], LDL GWAS2 [−0.55, 1.02], FC GWAS1 [−1.11, 1.70], FC GWAS2 [−1.06, 1.05]) are highligthed in blue (decrease) or red (increase).
(XLS)

Table S5 Pearson's correlations between analyzed parameters in GWAS1 versus GWAS2 RNAi screens.
(XLS)

Table S6 Regulation of LDLR upon knockdown of 35 different genes. Effects >2 standard deviations of untreated and mock (mRNA) or untreated and control siRNA (protein) treated cells are depicted in bold. * p<0.01, ** p<0.001, *** p<0.0001.
(XLS)

Table S7 Results of secondary assays for the respective siRNAs. Results for all siRNAs followed up in secondary assays. For GWAS1/2 siRNA-screening deviation of total cellular intensities is given (for other parameters see Table S4). LDLR mRNA and protein levels are gven as fold-change relative to control siRNA (for full results see Figures S4, S5 and Table S6). For localization and functional analysis of GFP-cDNAs, enzymatic determination of cellular cholesterol, FC quantification from HuH7 cells and definition of statistical thresholds, see Materials and Methods. Red, increase; blue, decrease. * p<0.01, ** p<0.001, *** p<0.0001.
(XLS)

Table S8 Results of candidate gene overexpression on cellular FC levels. For 29 genes where knockdown with ≥1 siRNA increased or reduced free cholesterol (FC) levels, consequences upon overexpression of a representative cDNA (carboxy-terminally linked to EGFP) on FC were analyzed (see Materials and Methods). Filipin staining was performed 24 h upon cDNA-transfection and fraction of GFP-positive cells ± SEM was determined. Ratios of FC in GFP-positive relative to non-expressing cells (within the identical dish) and ratios of FC in GFP-positive relative to mock-transfected cells are given as means ± SEM (n = 3–4 experiments). Effects were considered as significant (increase, red; decrease, blue) when a two-tailed Student's t-test resulted in p-values <0.01 in 2 (*) or 3–4 (***) experimental replicas.
(XLS)
