# Supplementary figures and images for: RNAi–Based Functional Profiling of Loci from Blood Lipid Genome-Wide Association Studies Identifies Genes with Cholesterol-Regulatory Function
Source: PLoS Genet. 2013 Feb 28;9(2):e1003338. doi: 10.1371/journal.pgen.1003338 (PMC3585126; doi:10.1371/journal.pgen.1003338)

Figure S1

A

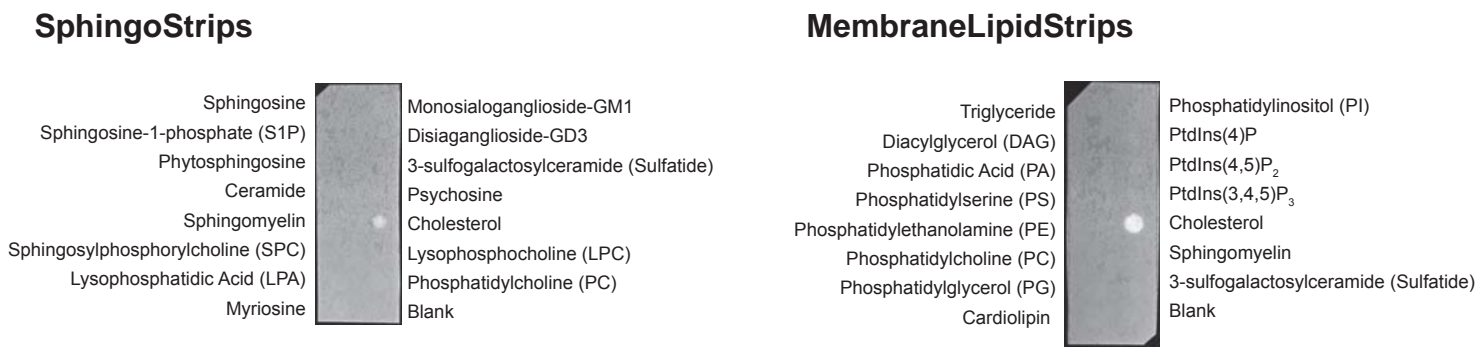

B

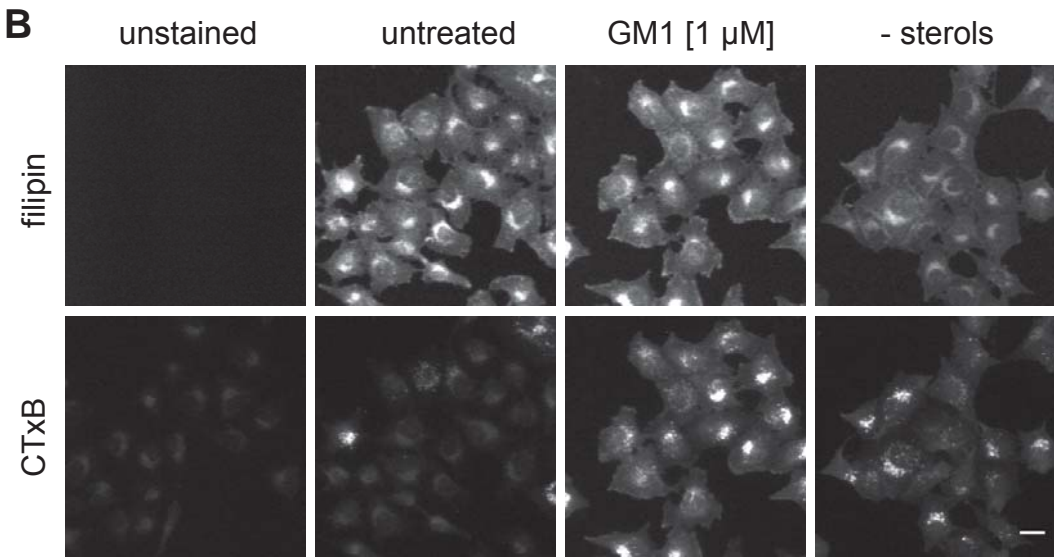

C

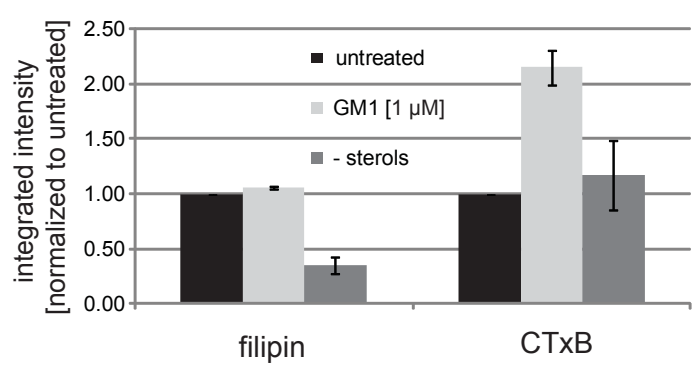

D

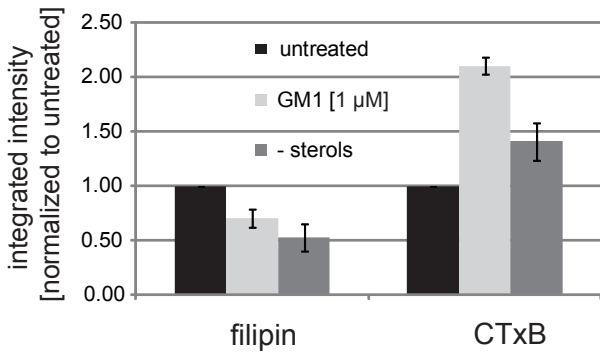

Supplement: Figure S1 — Assessing specificity of filipin III to detect free cholesterol. (A) Lipid strips representing 26 different lipid species were incubated for 30 min with 50 µg/ul filipin III in PBS and imaged with UV-light. (B–D) Hela-Kyoto cells were challenged by 15 min addition of 1 µM GM1 ganglioside to the medium or sterol-depletion by culture in DMEM/2 mM L-glutamine/0.5%BSA for 16 h followed by exposure for 45 min to 1% (w/v) hydroxyl-propyl-beta-cyclodextrin (HPCD) (− sterols) [30]. Images of filipin III or cholera toxin-AF568 (CTxB) stained cells were acquired on an automated widefield microscope. Bar = 20 µm. Total integrated intensities in perinuclear areas (filipin) or whole cell masks (CTxB) (C) or in a 100×100 pixel area at the cell peripheries (D) were quantified from 2 experimental replicas. Shown are means ± range of independent experiments. (PDF) [file pgen.1003338.s001.pdf]

Figure S2

**A** LDL-uptake assay

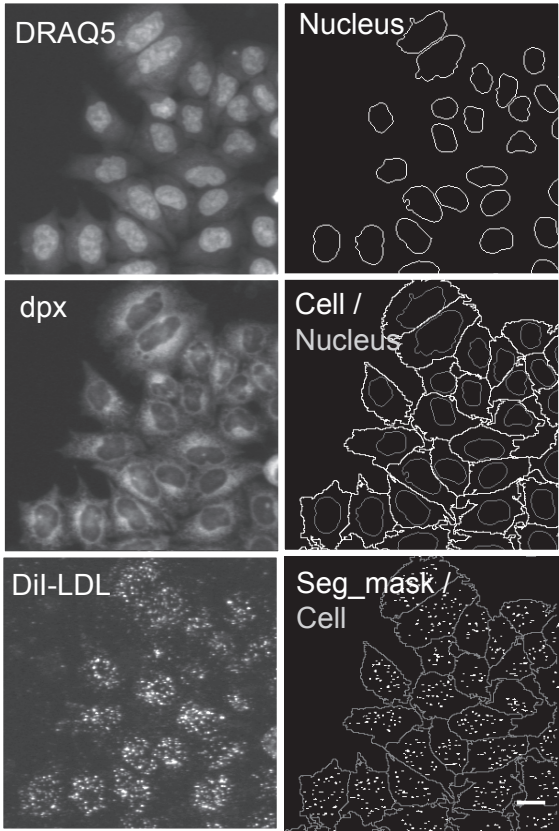

**B** free cholesterol (FC) assay

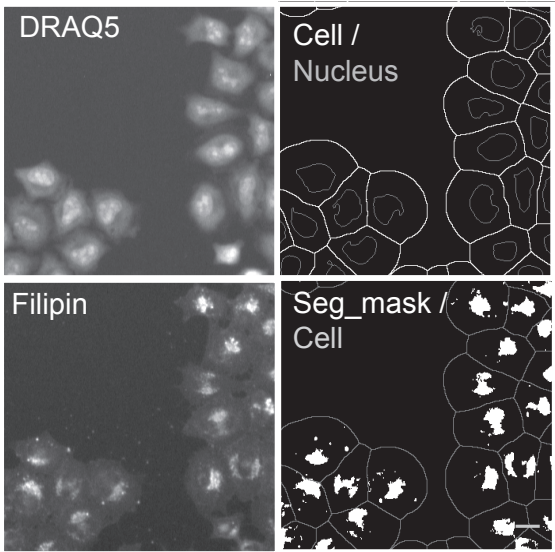

Supplement: Figure S2 — Pipelines for automated multi-parametric image analysis. Shown are representative images (left panels) acquired by automated fluorescence microscopy during RNAi-screening. (A) For measuring cellular LDL-uptake Hela-cells exposed to fluorescent DiI-LDL for 20 min at 37°C were fixed and stained for DRAQ5 (nuclei) and ER-Tracker dpx (endoplasmic reticulum). (B) For measuring FC, cells were stained with cholesterol-binding dye filipin III and DRAQ5 (for details see [30]). Masks (right panels) for quantification of DiI and filipin signal intensities from areas approximating whole cells and subcellular compartments were generated using Cellprofiler software (for details see Materials and Methods). (PDF) [file pgen.1003338.s002.pdf]

Figure S3

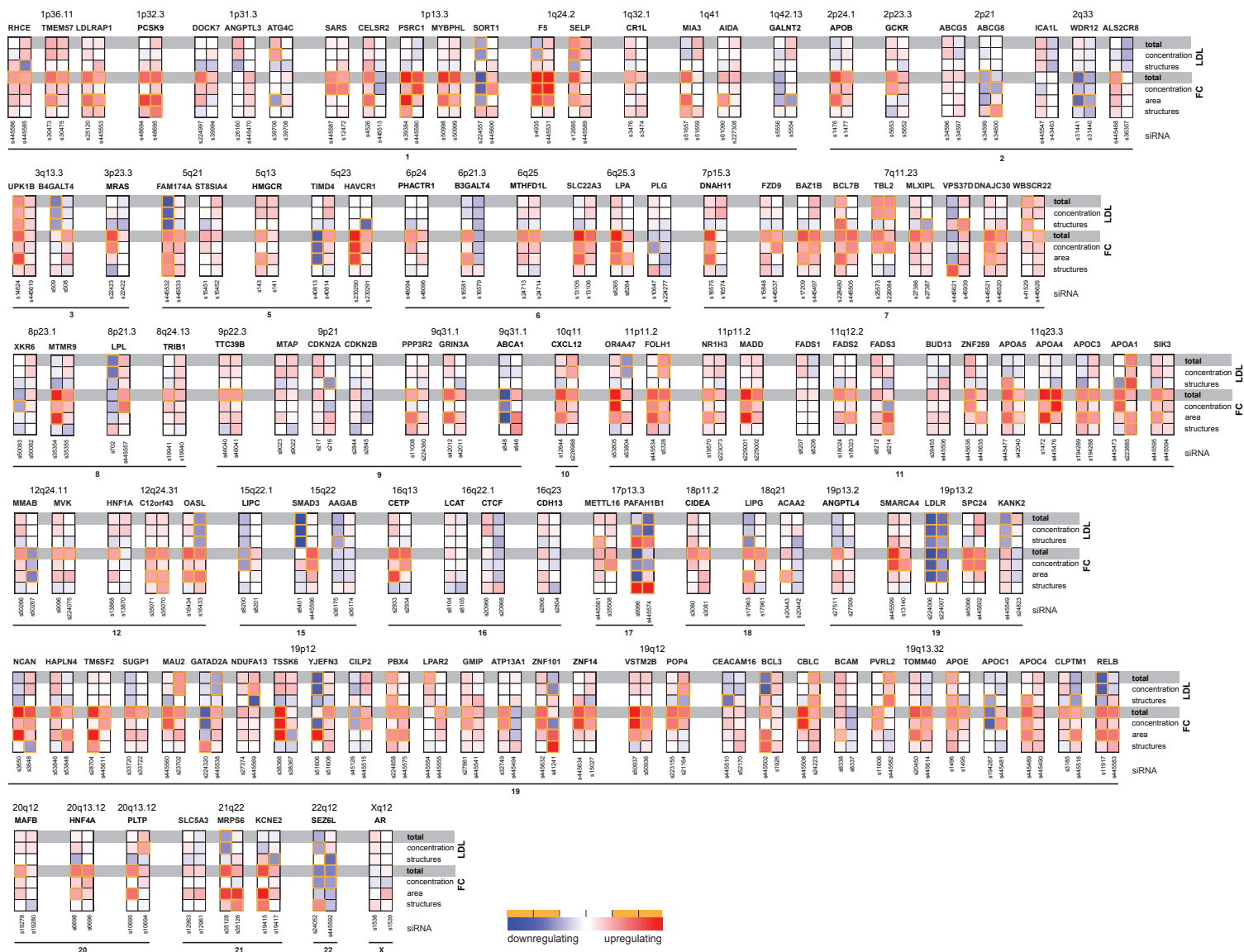

Supplement: Figure S3 — Multi-parametric analysis and clustering of 133 genes representing 56 lipid-trait/CAD/MI GWAS-loci. Functional consequences upon knockdown of each candidate gene were quantified from microscopic images with regard to seven phenotypic parameters: total cellular LDL-signal; LDL concentration and number of cellular structures; total free cholesterol (FC) signal; and FC concentration, area and number of cellular structures. For each of the 133 genes analyzed in this study, heatmaps of the two siRNAs causing the strongest effect on the parameters total cellular signal intensities (grey bar) are shown (see Table S4 for numeric data). Phenotypes (red, increasing; blue, decreasing) where mean effect size was above or below that of all 19 non-silencing control siRNAs are framed in orange. Genes are sorted according to chromosomal position. (PDF) [file pgen.1003338.s003.pdf]

Figure S4

A

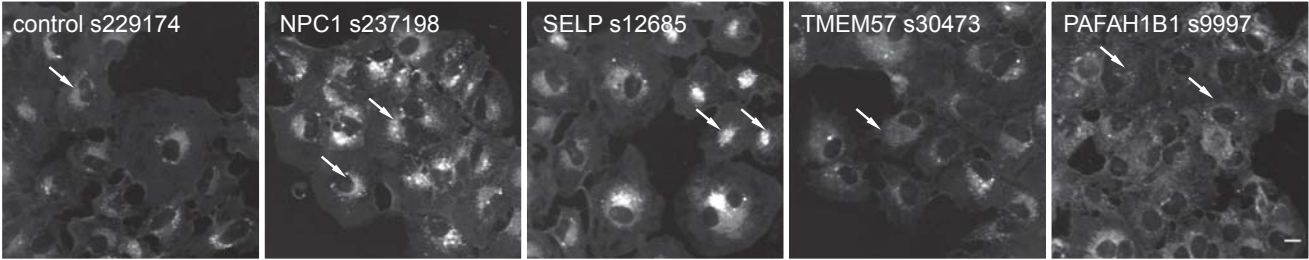

B

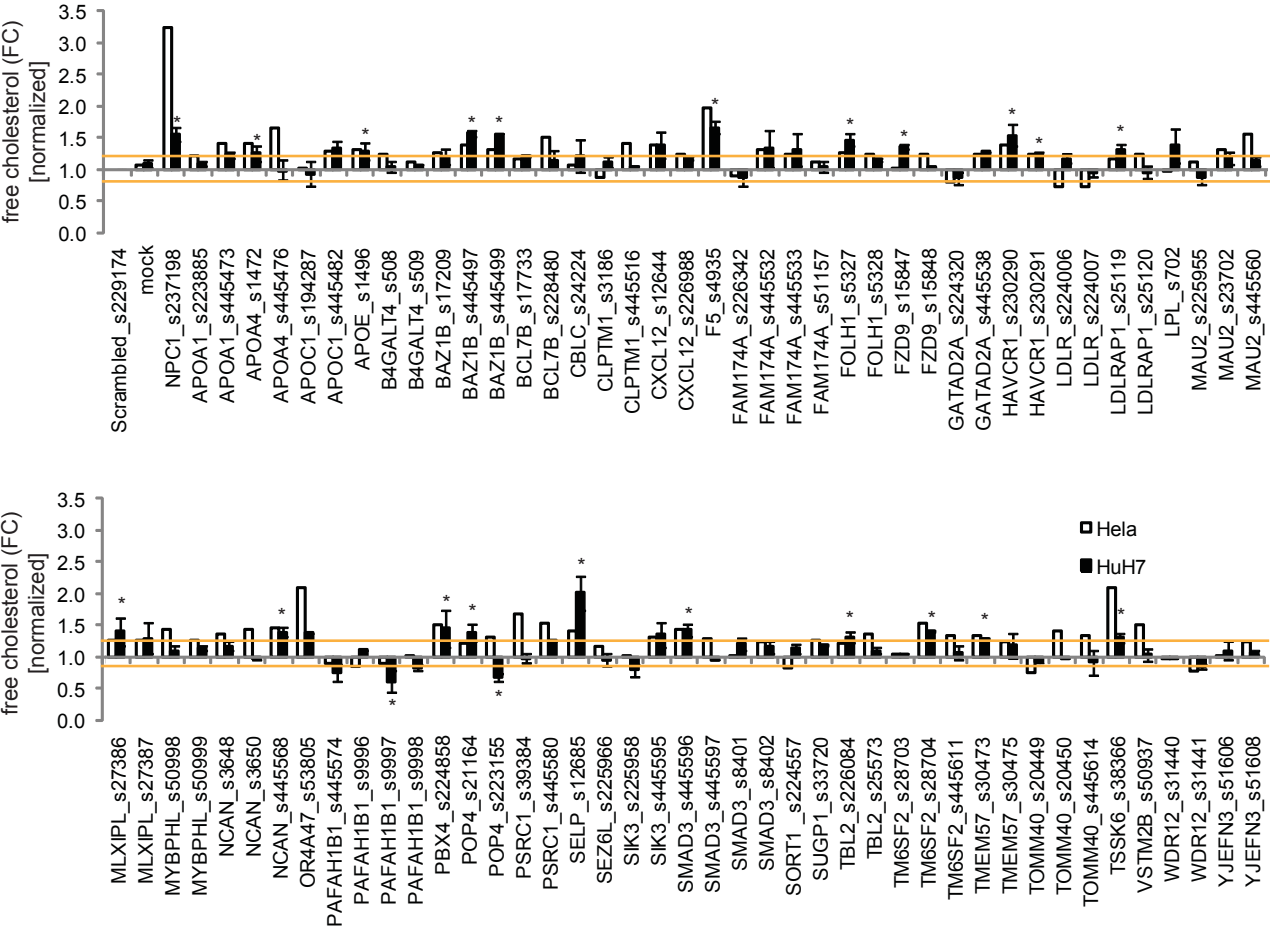

C

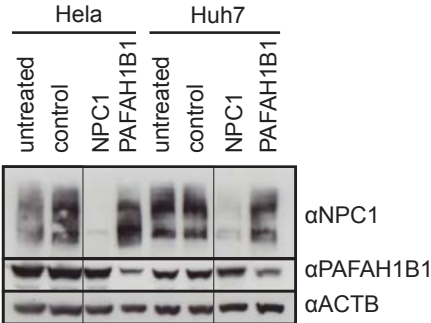

D

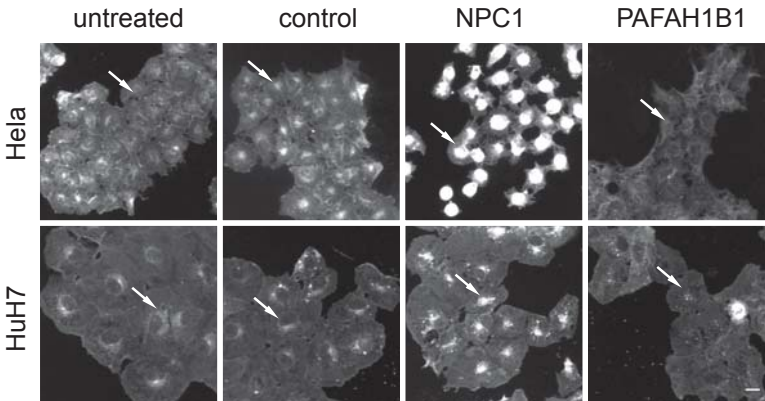

Supplement: Figure S4 — Analysis of free cholesterol (FC) levels in siRNA-treated HuH7 liver cells. HuH7 liver-derived cells were reverse siRNA-transfected for 72 h with 84 siRNAs targeting 42 selected candidate genes from GWAS-loci. (A) Representative images of cells treated with indicated siRNAs acquired by automated fluorescence microscopy during RNAi-screening. (B) Mean perinuclear total free cholesterol (FC) signals relative to control siRNA-treated cells in Hela-Kyoto (open bars) relative to HuH7 cells (filled bars). SiRNAs where mean functional effects in HuH7 cells exceeded upper or lower thresholds (indicated by orange lines) in ≥2 experimental replicates (by ≥2 standard deviations of negative controls) are highlighted by asterisks (see Table S7 for numeric data). (C,D) Comparison of protein knockdown efficiencies (C) and filipin signals (D) for two selected effector genes (NPC1, PAFAH1B1) in Hela-Kyoto and HuH7 cells. Note that at similar knockdown efficiencies functional effects were considerably less prominent in HuH7 relative to Hela-cells. Bars = 20 µm. (PDF) [file pgen.1003338.s004.pdf]

Figure S5

Plate 1

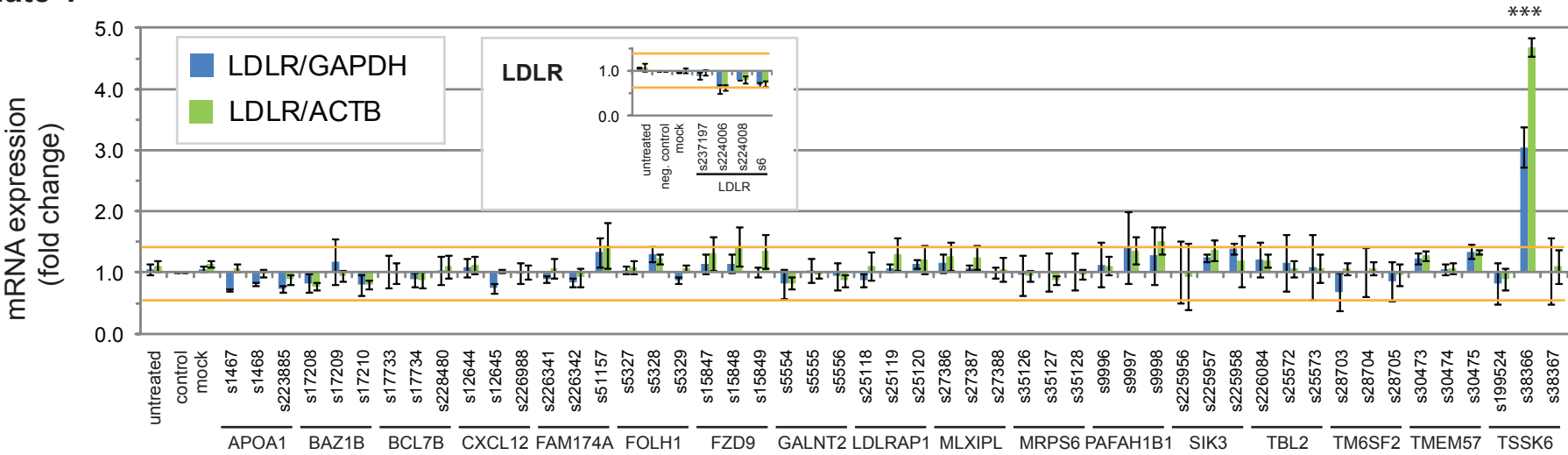

Plate 2

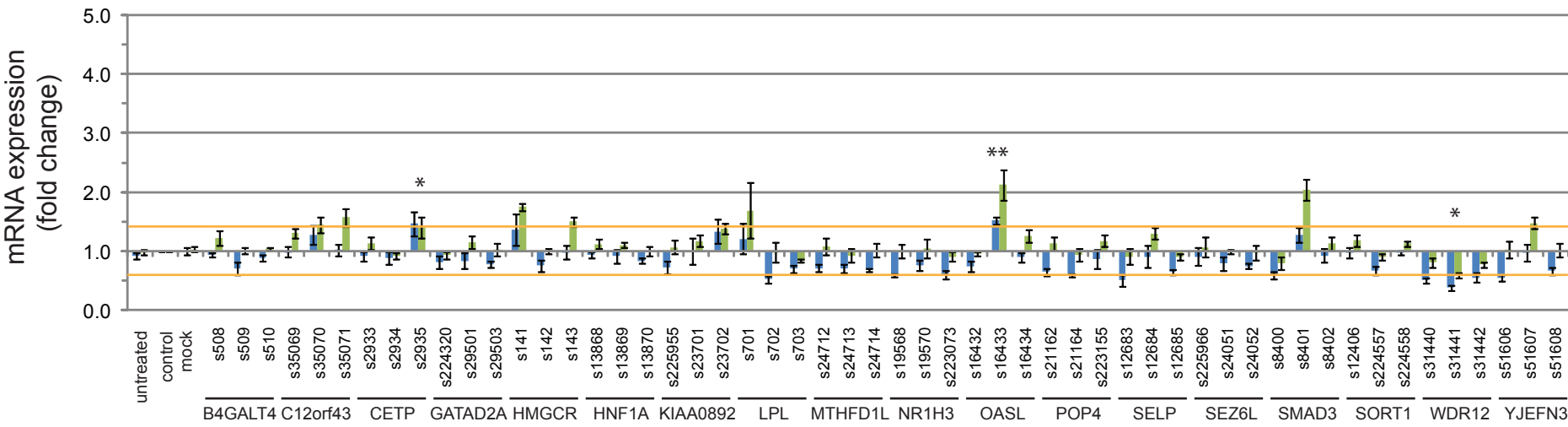

Supplement: Figure S5 — LDLR mRNA levels upon RNAi of 35 lipid-trait/CAD/MI associated genes. LDLR mRNA levels in Hela-Kyoto cells were assessed by qPCR upon knockdown of 105 siRNAs targeting 35 selected candidate genes from GWAS-loci. LDLR levels were normalized to housekeeping genes GAPDH (blue) and ACTB (green). For each siRNA the mean fold change ± SEM of 3–4 biological replicates is shown (see Table S6 for numeric data). Significance thresholds (≥2 standard deviations of negative controls; orange lines) and siRNAs reaching significant p-values in two-sided Student's t-test (*<0.01; **<0.001; ***<0.0001) are indicated. (PDF) [file pgen.1003338.s005.pdf]

Figure S7

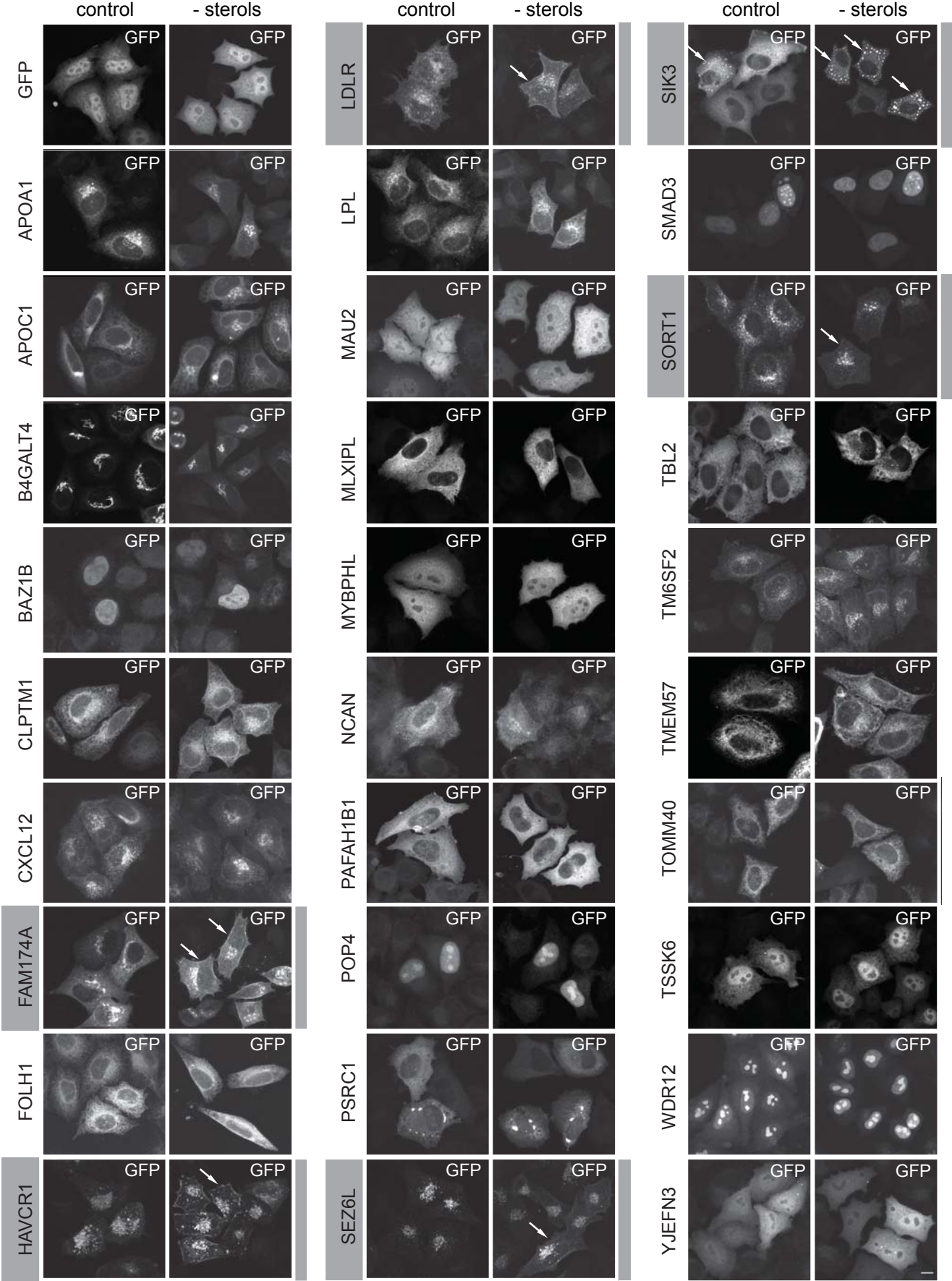

Supplement: Figure S7 — Subcellular localization of 29 GFP–tagged proteins encoding lipid-trait/CAD/MI associated genes. Hela-Kyoto cells were transfected for 24 h with cDNAs expressing indicated proteins carboxy-terminally linked to eGFP. Cells were cultured either under control or sterol-depleted conditions (by culture in DMEM/2 mM L-glutamine/0.5%BSA for 16 h followed by exposure for 3 h to 1% (w/v) hydroxyl-propyl-beta-cyclodextrin (HPCD) (− sterols) [30]. Shown are maximal projections of confocal stacks of representative cells. Proteins for which subcellular localization differed between control and sterol-depleted conditions are highlighted in grey and with arrows. Bar = 10 µm. (PDF) [file pgen.1003338.s007.pdf]
