# Supplementary material for: RNAi–Based Functional Profiling of Loci from Blood Lipid Genome-Wide Association Studies Identifies Genes with Cholesterol-Regulatory Function
Source: PLoS Genet. 2013 Feb 28;9(2):e1003338. doi: 10.1371/journal.pgen.1003338 (PMC3585126; doi:10.1371/journal.pgen.1003338)
Supplement: Figure S6 — LDLR protein levels upon RNAi of 35 lipid-trait/CAD/MI associated genes. LDLR protein levels in Hela-Kyoto cells were assessed by Western Blot upon knockdown of 105 siRNAs targeting 35 selected candidate genes from GWAS-loci. LDLR levels quantified from blots were compared either directly or normalized to housekeeping gene α-tubulin. Shown are results from 3 biological replicates (see Table S6 for numeric data). Arrows denote siRNAs that significantly (*<0.01; **<0.001; ***<0.0001) reduced (blue) or increased (red) LDLR protein levels above or below thresholds (≥2 standard deviations of negative controls). Bands from lysates where no reliable results could be obtained were crossed out and excluded from quantitative analysis. (PDF) [file pgen.1003338.s006.pdf]

Figure S6

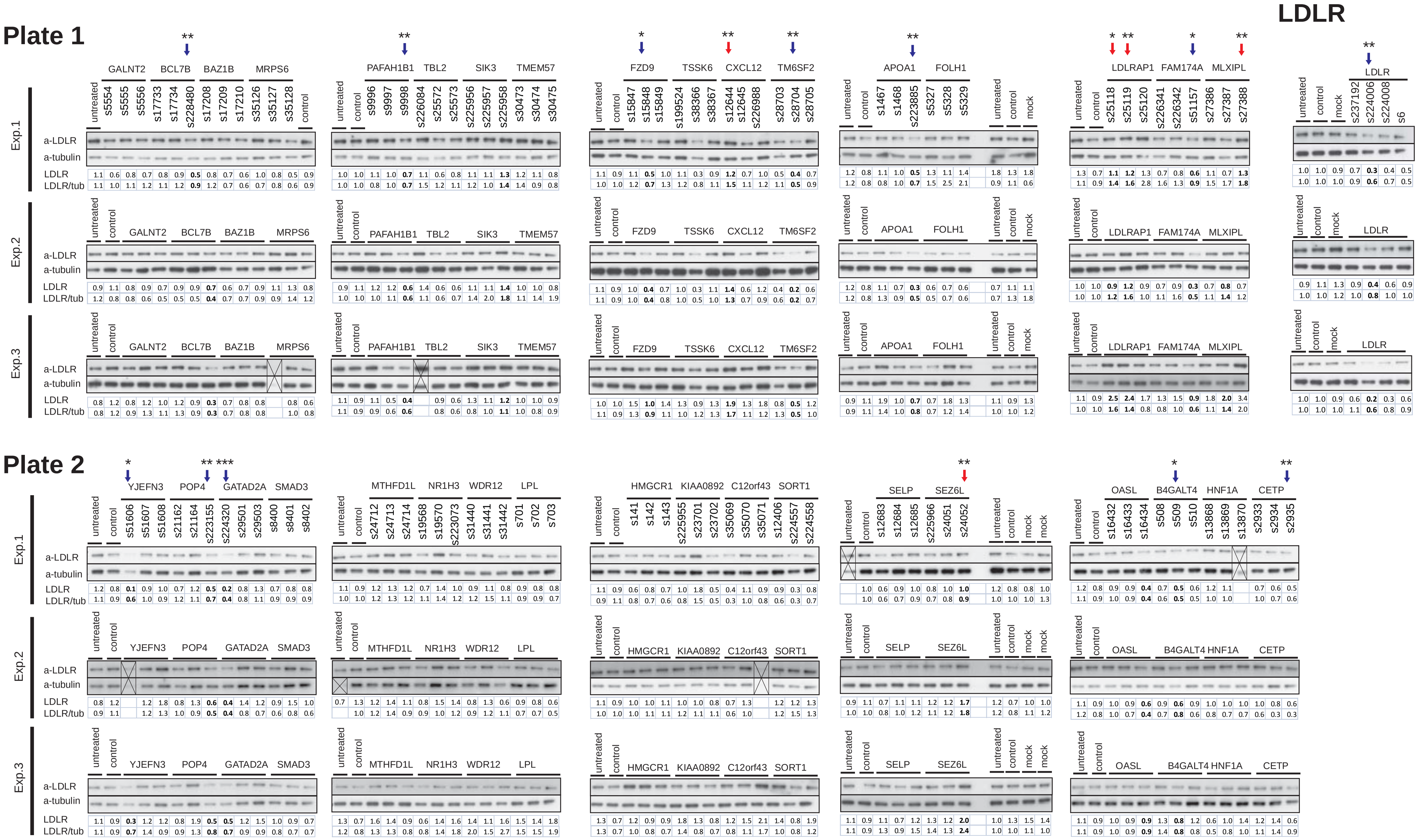

LDLR
Plate 1
Plate 2
Exp.1
Exp.2
Exp.3
a-LDLR
a-tubulin
LDLR
LDLR/tub
untreated
control
mock
GALNT2
BCL7B
BAZ1B
MRPS6
PAFAH1B1
TBL2
SIK3
TMEM57
FZD9
TSSK6
CXCL12
TM6SF2
APOA1
FOLH1
LDLRAP1
FAM174A
MLXIPL
YJEFN3
POP4
GATAD2A
SMAD3
MTHFD1L
NR1H3
WDR12
LPL
HMGCR1
KIAA0892
C12orf43
SORT1
SELP
SEZ6L
OASL
B4GALT4
HNF1A
CETP
